# Supporting the Development and Adoption of Automatic Lameness Detection Systems in Dairy Cattle: Effect of System Cost and Performance on Potential Market Shares

**DOI:** 10.3390/ani7100077

**Published:** 2017-10-08

**Authors:** Tim Van De Gucht, Stephanie Van Weyenberg, Annelies Van Nuffel, Ludwig Lauwers, Jürgen Vangeyte, Wouter Saeys

**Affiliations:** 1 Institute for Agricultural and Fisheries Research—ILVO, Technology and Food Sciences Unit, Burg, van Gansberghelaan 115, 9820 Merelbeke, Belgium; stephanie.vanweyenberg@ilvo.vlaanderen.be (S.V.W.); annelies.vannuffel@ilvo.vlaanderen.be (A.V.N.); jurgen.vangeyte@ilvo.vlaanderen.be (J.V.); 2 KU Leuven Department of Biosystems, MeBioS, Kasteelpark Arenberg 30 Box 2456, 3001 Leuven, Belgium; wouter.saeys@biw.kuleuven.be; 3 Institute for Agricultural and Fisheries Research—ILVO, Social Sciences Unit, Burg, van Gansberghelaan 115, 9820 Merelbeke, Belgium; Ludwig.lauwers@ilvo.vlaanderen.be; 4 Department of Agricultural Economics, Faculty of Bio-Engineering, Ghent University, Coupure Links 653, 9000 Gent, Belgium

**Keywords:** adoption rate, market share, willingness to pay, willingness to adopt, automatic lameness detection, discrete choice experiment

## Abstract

**Simple Summary:**

Most prototypes of systems to automatically detect lameness in dairy cattle are still not available on the market. Estimating their potential adoption rate could support developers in defining development goals towards commercially viable and well-adopted systems. We simulated the potential market shares of such prototypes to assess the effect of altering the system cost and detection performance on the potential adoption rate. We found that system cost and lameness detection performance indeed substantially influence the potential adoption rate. In order for farmers to prefer automatic detection over current visual detection, the usefulness that farmers attach to a system with specific characteristics should be higher than that of visual detection. As such, we concluded that low system costs and high detection performances are required before automatic lameness detection systems become applicable in practice.

**Abstract:**

Most automatic lameness detection system prototypes have not yet been commercialized, and are hence not yet adopted in practice. Therefore, the objective of this study was to simulate the effect of detection performance (percentage missed lame cows and percentage false alarms) and system cost on the potential market share of three automatic lameness detection systems relative to visual detection: a system attached to the cow, a walkover system, and a camera system. Simulations were done using a utility model derived from survey responses obtained from dairy farmers in Flanders, Belgium. Overall, systems attached to the cow had the largest market potential, but were still not competitive with visual detection. Increasing the detection performance or lowering the system cost led to higher market shares for automatic systems at the expense of visual detection. The willingness to pay for extra performance was €2.57 per % less missed lame cows, €1.65 per % less false alerts, and €12.7 for lame leg indication, respectively. The presented results could be exploited by system designers to determine the effect of adjustments to the technology on a system’s potential adoption rate.

## 1. Introduction

Current dairy research focuses on the development of technologies to detect health problems such as lameness, mastitis, and metabolic disorders [1] to improve farm profitability, animal health, and animal welfare. For the manufacturer, a high adoption rate is necessary to cover development and marketing costs. Potential adoption rates should therefore be estimated in advance to design systems that are useful for the farmer, but also commercially feasible [2]. 

The adoption of a health monitoring system depends on the usefulness that farmers attach to a system with specific characteristics. Possibly, farmers’ preferences could be diverse for different types of technology and hence lead to different potential adoption rates. As emerging technologies are not yet used in practice, no real market shares are available to demonstrate the effect of alternating system performances or costs. A simulation of potential market shares could provide an insight into these effects, and could be performed based on a utility model describing the usefulness that farmers attach to different technologies with specific characteristics. Such a utility model can be constructed based on the choice behavior of farmers as explored using discrete choice experiments (DCE). Revealing the preferences of the end user could support a more focused development, as system developers know what farmers expect and can take these expectations into account during development. 

Lameness is an important health and welfare problem with major consequences for productivity and farm profitability [3,4], but current detection methods are time consuming and subjective. Therefore, many research initiatives have focused on creating systems to detect lameness automatically and objectively. The most used technologies are camera systems [5,6], walkover systems [7,8], and cow-attached systems [9,10]. Except for StepMetrix™ (Boumatic, MA, WI, USA), none of these systems are already commercially available. Since the aforementioned research prototypes may be further developed into commercial applications, it would be useful to know the effect of important system characteristics on their potential market share. Such characteristics could be the system cost, the rates of undetected lame cows and false alarms, the ability to indicate which leg is lame, and how much earlier a lame cow can be detected. As it is unclear how much earlier a lameness case could be detected by using an automatic lameness detection system [11], this characteristic cannot easily be accounted for in market share calculations. For certain system types, the system cost may depend on the herd size, thus requiring a general cost characteristic when surveying farmers’ preferences. In a previous study, we determined the importance of several aforementioned automatic lameness detection system characteristics using a DCE [12], but information about the path that will maximize the chances for adoption by the sector has not yet been provided.

The goals of this study were therefore to: (1) investigate the effect of an improved lameness detection performance on the uptake of such technologies in dairy practice; (2) assess the influence of system costs on the potential adoption rate; and (3) demonstrate the usefulness of discrete choice experiments to define development goals to improve the adoption potential of existing prototypes.

## 2. Materials and Methods

### 2.1. Experimental Approach

We performed a DCE to investigate which system characteristics of an automatic lameness detection system are considered important by Flemish dairy farmers [12]. Farmers had to choose their preferred option from four options: a system attached to the cow, a walkover system, a camera system, and visual detection without the help of a sensor system. In Table 1, the system characteristics and their possible levels as used in the DCE are summarized. With the information in Table 1, choice sets consisting of specific combinations of system characteristic levels were created for each sensor system. An example is given in Table 2.

The DCE was embedded in an online survey that was active for two months. Each respondent was shown two blocks of four choice sets each; one before providing extra information on lameness consequences and one after, allowing us to find out whether extra information on lameness consequences influenced farmers’ decision behavior. In addition, general questions concerning the farm and current practices were asked to determine socio-demographic characteristics describing the farm. In total, 135 responses from Flemish dairy farmers (originating from the northern part of Belgium) were gathered, totaling up to 1080 answered choice sets. Socio-demographic characteristics of the respondents were found to correspond fairly well with Flemish averages. For more details on the gathered responses, the reader is referred to [12]. 

Farmers’ choices were modeled in a utility model describing the usefulness that a farmer attached to each available option in the choice set. In this model, the system cost, percentage false alarms, percentage missed lame cows, and ability of the system to indicate which leg is lame had a significant influence on the farmers’ perceived usefulness of automatic lameness detection systems. The reader is referred to [12] for more details on the modeling process. The resulting utility model was used as a basis to simulate potential market shares from the gathered responses and was given by (Equation (1)):

(1) $$\begin{array}{ll} U_{i} & =\beta_{0i}+\beta_{1}*missedLame+\beta_{2}*falseAlarms+\beta_{3}*\cos t\\ & +\beta_{4}*indicateLeg+\beta_{IMPLAMENESS}*impLameness+\\ & \beta_{CALFINSEMD1}*calfInsemd1+\beta_{CALFINSEMD2}*calfInsemd2+\\ & \beta_{ESTDET}*estDet1+\beta_{BEFOREAFTER}*beforeafter \end{array}$$

where *U_i_* is the utility (usefulness, dimensionless value relative to the opt-out option with utility zero) of option *i*, *β*_0*i*_ is the model constant of option *i*, *missedLame* is the percentage missed (undetected) lameness cases, *falseAlarms* is the percentage false alarms, *cost* is the system cost, and *indicateLeg* is the ability to indicate which leg is lame, each with its respective coefficient *β*_1_, *β*_2_, *β*_3_, and *β*_4_. The importance that the farmer attached to lameness on a scale from 1 to 10 (*impLameness*), the interval calving to first insemination (*calfInsemd*1 and *calfInsemd*2) on the farm, whether there was an automatic estrus detection system present on the farm (*estDet*), and whether the farmer received extra information about the consequences of lameness before making a choice (*beforeafter*), were retained in the model, each with their corresponding coefficient estimate (*β*).

Equation (1) was used to calculate the average utility of the 135 farmers for one set of system characteristics for each technology and for visual detection. The market share (%) of each technology and visual detection, given the used system characteristics, could be calculated as a ratio between the average utility of one technology divided by the sum of the average utilities of all four options (the three technologies and visual detection) (Equation (2)):

(2) $$\mathrm{Market}\;\mathrm{share}\;\mathrm{system}\;i=\frac{\exp(\frac{1}{N}\sum_{n=1}^{N}U_{i,n})}{\sum_{m=1}^{M}\exp(\frac{1}{N}\sum_{n=1}^{N}U_{m,n})}$$

where *N* is the number of respondents (135), *M* is the number of systems including visual detection (4), and *U_i,n_* is the utility attached to system *i* by farmer *n*. If the average utility that farmers attach to each of the four systems would be equal, all systems would obtain a market share of 25%. The effect of altering system characteristics on the market share was assessed by comparing simulated market shares for two parametrizations: one where the performance of the detection systems was gradually improved and one where the system price was gradually reduced.

### 2.2. Effect of System Performance on the Potential Market Share

To evaluate the effect of an improved system performance on the market share, the percentage false alarms and the percentage missed lame cows were decreased gradually, whereas the cost and the ability to identify which leg is lame were kept constant for all systems. The following combinations of percentage false alarms and percentage missed lame cows were simulated: (simulation 1) 30% false alarms and 18% missed lame; (simulation 2) 24% false alarms and 12% missed lame; (simulation 3) 18% false alarms and 9% missed lame; (simulation 4) 12% false alarms and 6% missed lame; and (simulation 5) 6% false alarms and 3% missed lame. These five simulations were performed in combination with four situations with different cost values and lame-leg-indication: (1) cost of €20 and no indication which leg is lame; (2) cost of €20 per cow per year with indication which leg is lame; (3) cost of €35 per cow per year without indication which leg is lame; and (4) cost of €35 with indication which leg is lame. The example system costs were chosen based on an expected lameness cost of €53 per cow per year [13], and on the assumption that the cost of a detection system should not exceed the proportion of the lameness cost that can be avoided to be economically profitable for the farmer. As the potentially avoided losses are currently still unknown, the two system costs, €20 and €35 per cow per year, were chosen as a deemed realistic cost for automatic lameness detection systems ready for adoption in practice. All simulations were performed twice: first for the situation before the farmer received extra information about the consequences of lameness (i.e., milk loss, underestimation prevalence, financial losses, etc.) (*beforeafter* = 0 in Equation (1)), and again for the situation after receiving extra information (*beforeafter* = 1 in Equation (1)). In total, 40 simulations were performed.

### 2.3. Effect of System Cost on the Potential Market Share

Next, the cost of all systems was gradually reduced to evaluate the effect of the system cost on potential market shares. System costs ranged from €75 to €15 and decreased in steps of €5 for all systems simultaneously. During the simulation, all other attributes (% false alerts, % missed alerts, lame-leg-indication) were kept constant for all systems. Two simulated system performances (20% missed lameness cases combined with 10% false alerts and 10% missed lameness cases combined with 5% false alerts) were chosen to represent a mediocre and a rather well performing system, respectively. Simulations for four different system performance levels were performed: (simulation 1) 5% false alarms and 10% missed lameness cases without indication which leg is lame; (simulation 2) 5% false alarms and 10% missed lameness cases with indication which leg is lame; (simulation 3) 10% false alarms and 20% missed lameness cases without indication which leg is lame; and (simulation 4) 10% false alarms and 20% missed lameness cases with indication which leg is lame. Similar to the previous section, all simulations were performed for the situation before and after the farmer received extra information. Hence, in total, 104 simulations were performed.

### 2.4. Willingness to Pay for Improved System Characteristics

Willingness to pay (WTP) was calculated as a measure for the amount of money that a farmer is willing to spend for a certain improvement in one of the system characteristics. WTP is calculated as the ratio of two system characteristic coefficient estimates mentioned in Equation (1). Hence, WTP was calculated for each system characteristic by dividing the coefficient value of the respective characteristic *β_j_* by the coefficient value for the system cost (*β*_3_)

(3) $$WTP_{j}=\frac{\beta_{j}}{\beta_{3}}$$

where *j =* 1 for percentage missed lame cows, *j =* 2 for percentage false alerts, and *j =* 4 for ability to indicate which leg is lame. Consequently, WTP is expressed in € per % point improvement for percentage missed lame cows and percentage false alerts, and as an amount of money (€) if lame leg indication is present. 

### 2.5. Usefulness of the Discrete Choice Experiment for Further Technology Development

**Figure 1:**
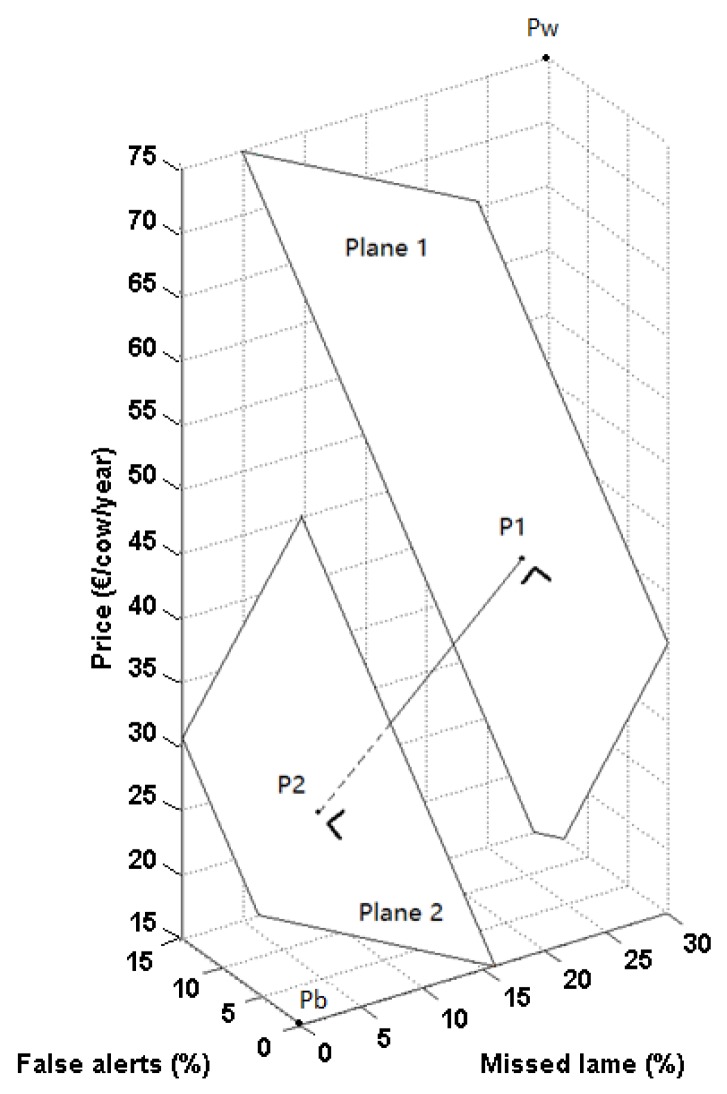
Schematic representation of the combination of system characteristics of the desired system. Pw represents the combination of characteristic levels for the system considered worst, with 30% missed lame cows, 15% false alerts, and a cost of €75/cow/year. Pb represents the system that is considered best, with 0% missed lame cows, 0% false alerts, and the lowest cost (€15/cow/year). P1 is the point representing the example current characteristic levels with plane 1 containing all system characteristic combinations with utility equal to P1. Plane 2 contains all system characteristic combinations that have the desired utility, and from which the most feasible option can be selected. P2 represents the combination with the smallest possible alterations to the current characteristics needed to reach the desired utility value for a future system.

The potential adoption rate of a lameness detection system can be enlarged by altering the system characteristic levels in such a way that the utility of the system increases. The utility model (Equation (1)) can be used to provide guidelines for system developers to increase the adoption potential of their system. These guidelines can be visualized by presenting the utility attached to specific combinations of system characteristic levels in a 3D plot. First, possible combinations of automatic lameness detection system characteristic levels (% missed lame cows, % false alerts, cost per cow per year and the ability of the system to indicate which leg is lame) lying within the intervals mentioned in Table 1 were listed. Visualizations were composed of 3D-plots where each point of the 3D-grid represents one possible combination of system characteristic levels (Figure 1). Separate plots were made for systems with or without lame leg indication. The color of each plotted point was determined according to the size of the mean utility calculated using Equation (1). 

Within the 3D-plots, planes of points with similar utility values (hence similar color) can be defined, creating a stack of parallel iso-utility planes. This allows developers to identify which point in the 3D-grid belongs to the characteristic level of their current system. Consecutively, a desired utility value for the future system can be proposed. This implies that the respective plane containing the combination of desired characteristics is also known. Two planes can thus be defined: a first one containing the combination of characteristic levels of the current system (Figure 1, plane 1), and a second one containing the combination of characteristic levels of the desired improved system (Figure 1, plane 2). The latter can be defined by identifying the system characteristic levels that provide the desired utility value at the shortest distance from the current characteristic levels (P1). This point is the intersection point of the second plane and a line perpendicular to the first plane through the point with the current system characteristic levels (Figure 1, P1).

Using these visualizations, guidelines can be defined to indicate a direction to which further development should be focused to reach the desired result. An arbitrarily chosen example of a system that would benefit from improvement was used to demonstrate how such guidelines can be defined. A current system performance of 27% missed lame cows, 13% false alerts, a cost of €41 per cow per year, no lame leg identification, and a utility value of −1.20 was used as an example. Two example desired systems (i.e., improved versions of the example current system) were defined: the first system should have a utility value of 0, indicating that the system would incur equal usefulness compared to visual detection. The second system was defined by a utility value of 0.5. For each system, a combination of system characteristic levels that fulfills the prerequisites was defined for the case of a system attached to the cow. Both systems with and without lame leg identification were assessed for the future system configuration. 

## 3. Results

### 3.1. Effect of Detection Performance on Potential Market Share

**Figure 2:**
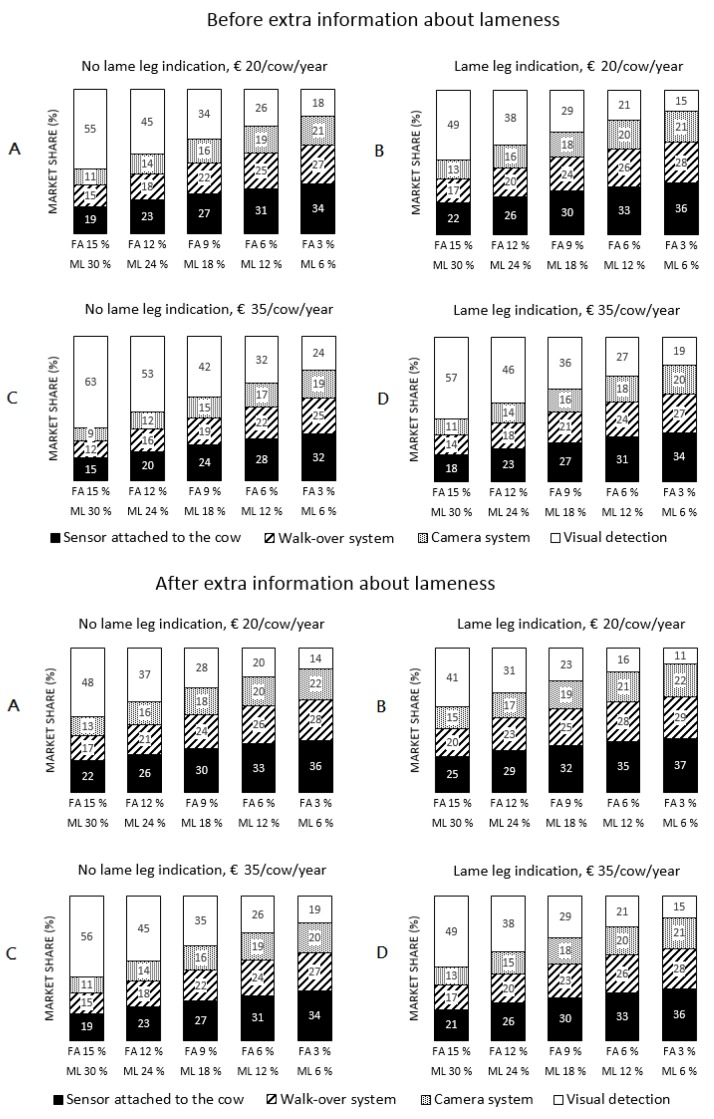
Simulated market shares (%) of the four detection systems after reducing the percentage missed lame cows (ML) and the percentage false alarms (FA) for four different situations before and after the farmer received extra information about lameness. (**A**) (situation 1), system cost €20/cow/year, no indication which leg is lame; (**B**) (situation 2), system cost €20/cow/year, indication which leg is lame; (**C**) (situation 3), system cost €35/cow/year, no indication which leg is lame; (**D**) (situation 4), system cost €35/cow/year, indication which leg is lame.

In Figure 2, the simulation results for the effect of the system performance on the potential market share are shown. The market share of automatic detection systems was 2% larger on average when the system can indicate which leg is lame compared to a similar system without this feature. This was both the case for the situation with the cheaper systems (A & B) and the situation with the more expensive systems (C & D). The market shares of systems with a similar performance were smaller for more expensive systems (€35) compared to cheaper systems (€20). The market share of visual lameness detection was about 6% lower on average when comparing similar sensor configurations in similar situations before and after the farmer received the extra information. Market shares increased by 3% on average when the percentage missed lame cows and the percentage false alarms were decreased by 6% and 3%, respectively.

Within each situation in Figure 2, the market shares of the automatic systems increased as the detection performance improved. In general, the market share of visual detection was more than 50% in three of the four situations in the sensor configuration with the worst detection performance before the farmer received extra information. Visual detection was hence preferred over automatic detection systems. This market share decreased rapidly as the detection performance improved, resulting in the completely opposite situation when automatic systems had a high detection performance. The market shares within similar situations (A & C, and B & D), but with a different cost, were also different. Situations with more expensive systems had slightly lower market shares compared to the situations with cheaper systems. Although the cost of the more expensive system (€35/cow/year) was almost double that of the cheaper system (€20/cow/year), the market shares were only a few percentage smaller for the more expensive system when comparing similar performances between the situations. Systems capable of indicating which leg is lame realized market shares that were about 2% higher on average for each sensor system compared to similar systems that cannot indicate which leg is lame.

The fact that the system performance of each sensor was changed simultaneously led to proportional changes between sensor systems in all simulations. A sensor attached to the cow had the biggest market share, followed by walkover systems and camera systems. A camera system with lame leg indication and a cost of €20 should not exceed a maximum of 12% missed lame cows and 6% false alarms in order to reach a similar market share as a walkover system with lame leg indication and a cost of €20 that had 24% missed lame cows and 12% false alarms. 

### 3.2. Effect of System Cost on Potential Market Share

**Figure 3:**
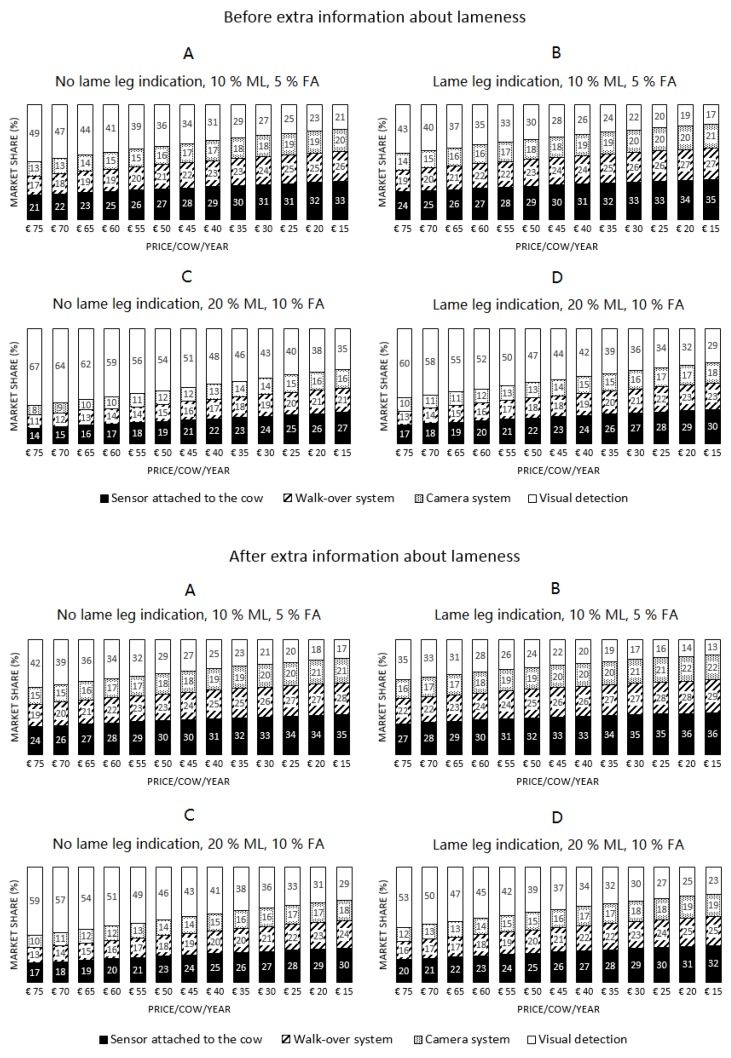
Simulated market shares (%) of the four detection systems after reducing the system cost for all systems in four different situations before and after the farmer received extra information. (**A**) (situation 1), 5% false alarms, 10% missed lame cows, no indication which leg is lame; (**B**) (situation 2), 5% false alarms, 10% missed lame cows, indication which leg is lame; (**C**) (situation 3), 10% false alarms, 20% missed lame cows, no indication which leg is lame; (**D**) (situation 4), 10% false alarms, 20% missed lame cows, indication which leg is lame.

In Figure 3, the influence of the system cost on potential market shares is presented. First, it should be noted that all presented results should be interpreted with care, as several assumptions that may not be valid in reality were made during the simulation (e.g., simultaneous changes for all automatic systems). In general, market shares of the automatic detection systems increased clearly with a decreasing system cost. Each system’s market share increased by about 0.75 percent on average per cost difference of €5. Market shares were larger when the system performance (% missed lame and % false alerts) was better, and slightly larger (2% on average) for systems capable of indicating which leg is lame compared to the same system without this feature. Market shares were slightly higher for equal system configurations in similar situations after the farmer received extra information about lameness.

The market shares in Figure 3 changed only slightly with each step in cost reduction. However, for large cost differences, these small changes added up to quite large differences. Visual detection clearly lost popularity when automatic systems became cheaper. Also, similar results compared to the first part were found: systems that could indicate which leg was lame had higher market shares than those that didn’t have this feature, but the difference was often only one percent. When comparing the evolution between situations with similar systems, but different detection performances (Figure 3A,C and Figure 3B,D), it was clear that the better performing systems had higher market shares. Similar to the results in Figure 2, the system attached to the cow had the highest market share of all automatic systems, followed by the walkover system. 

### 3.3. Willingness to Pay for Improved System Characteristics

Based on the variable coefficients in Equation (1), farmers were willing to pay €2.57 more per % point less missed lame cows, €1.65 more per % point less false alerts, and €12.7 more if the system was capable of indicating which leg is lame. This means that when the percentage missed lame cows of a system with a specific performance configuration is reduced by one percent, the system cost can increase simultaneously by €2.57 in order for the new system to reach an equal usefulness for the farmer and hence an equal market share compared to the original system. That is, if the percentage false alerts and the ability to indicate which leg is lame remain constant. For example, if a system with 27% missed lame cows, 13% false alerts, no indication which leg is lame, and costing €41 per cow per year, is altered to a system with 27% missed lame cows, 12% false alerts, and no indication which leg is lame, the system cost can increase to €42.65 per cow per year while still reaching the same utility for the farmer. If the same system is altered to a system with 26% missed lame cows, 12% false alerts, and no indication which leg is lame, the system cost can increase to €45.22 per cow per year, and still reach the same utility for the farmer. 

### 3.4. Defining Development Goals for Detection Performance

**Figure 4:**
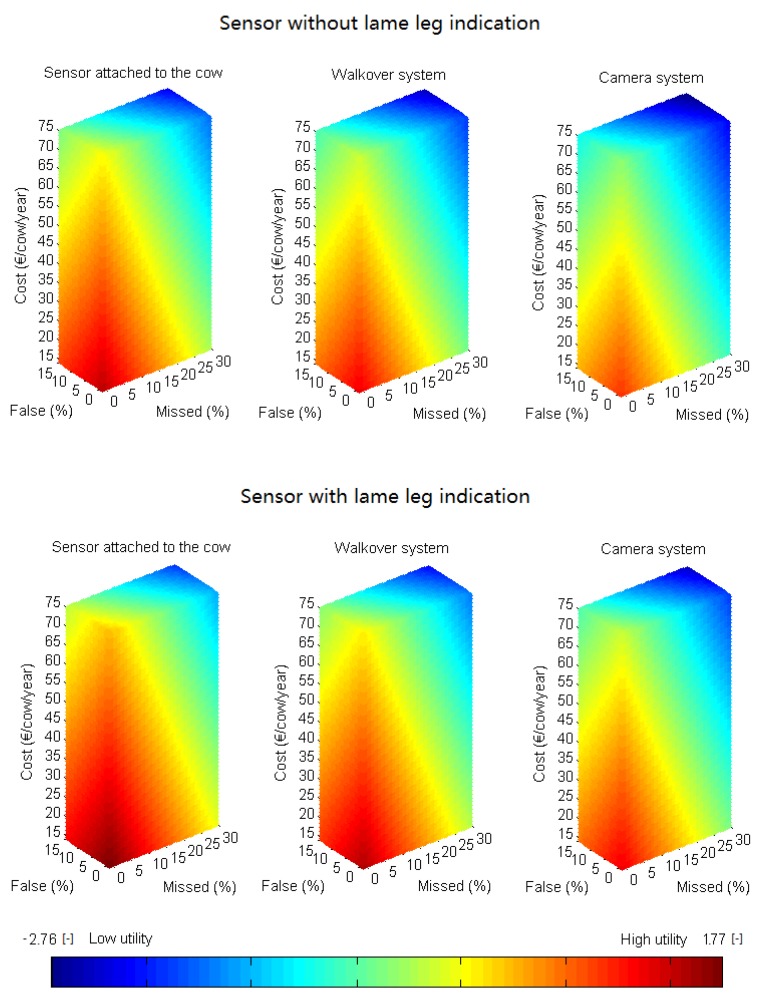
Visual representation of possible combinations of % missed lame cows, % false alerts, cost per cow per year, and the ability of the system to indicate which leg is lame, separated per sensor technology (sensor attached to the cow, walkover system, camera system). Planes with similar colors indicate equal utility values. Utility values (dimensionless, respective to the opt-out option) vary from low utility (dark blue) to high utility (dark red).

In Figure 4, visualizations of the utility of specific system characteristic combinations are represented per technology type and separated for systems with or without lame leg indication. Planes with equal colors indicate equal utility values between sensor systems. Systems with lame leg indication generally had higher utility values and hence more warm colors (red, yellow) and less low utility values (dark blue). Utility differences between technology types were clearly visible, as a sensor attached to the cow had higher utility values, visible as more warm colors, compared to the other technologies. Similarly, utility values were generally higher for walkover systems compared to camera systems. A utility value of 0 indicates equal usefulness compared to visual detection, and was colored in yellow. As such, only a rather small part of each figure involves utility values higher than that of visual detection, indicating the need for high detection performance and low system costs to achieve high adoption rates for automatic lameness detection systems.

**Figure 5:**
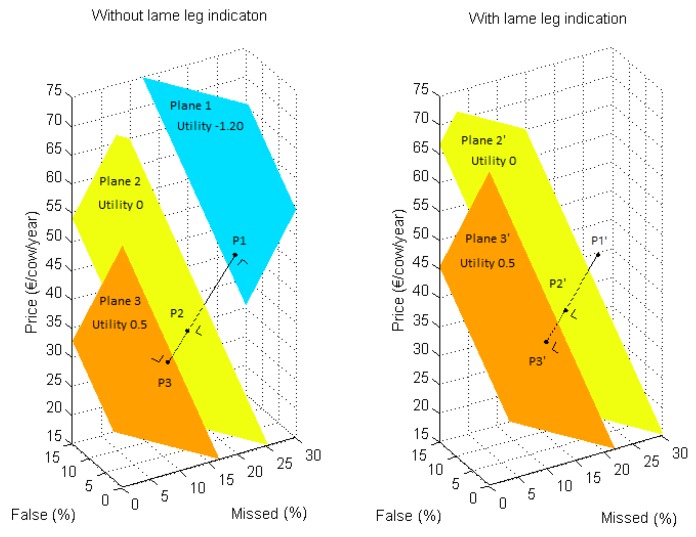
Visualization of the current system performance (P1), the desired system performance for utility value 0 (P2), and the system performance for a system with utility 0.5 (P3) for a system without lame leg indication attached to the cow (left figure). Plane 1 combines different combinations of characteristic levels with a utility of −1.20, whereas plane 2 and plane 3 combine system performances with a utility of 0 and 0.5, respectively. System performances for future systems with lame leg indication are presented on the right (P2′ and P3′). P1′ was therefore defined as a fictitious current system performance with the same 3D position (thus same % missed lame cows, % false alarms and price) as P1. Equal colors indicate equal utility values.

In Figure 5, the development goals to improve the example current system performance are presented visually for configurations with and without lame leg indication for the case of a sensor attached to the cow. Three points were defined: one representing the utility of the current system configuration (P1), one representing the system configuration for utility value 0 (P2), and one representing the system configuration for utility value 0.5 (P3), each lying within its respective plane of other combinations with equal utility values (plane 1, plane 2, and plane 3, respectively). Each combination of system characteristics (P2, P3) was found by determining the intersection point of the desired utility planes with the line through P1 (current system characteristics combination) perpendicular to plane 1. Hence, the combination of system characteristics that meets the desired utility with the smallest possible alterations (smallest spatial distance) to the current characteristic levels was found. The desired system performance with utility 0 was formed by a combination of 14% missed lame cows, 5% false alerts, and a cost of €36 per cow per year for a sensor system without lame leg indication (P2). The configuration for a system with utility 0.5 was 9% missed lame cows, 1% false alerts, and a cost of €34 per cow per year without lame leg indication. If the system could identify which leg was lame, the prerequisites for the other system characteristics became less strict. In this case, utility 0 was already reached with a system with 17% missed lame cows, 7% false alerts, and a cost of €37 per cow per year (P2′). Utility value 0.5 was obtained for 12% missed lame cows, 3% false alerts, and a cost of €35 per cow per year (P3′). 

## 4. Discussion

### 4.1. Automatic Systems Have to Become Cheaper and More Performant to Be Preferred over Visual Detection

The results of this study emphasized the effect of the cost and performance of automatic lameness detection systems on their potential market shares. When automatic systems become cheaper, visual lameness detection will become less and less popular in favor of the automatic systems, as farmers’ willingness to adopt increases. Similarly, if the detection performance of automatic detection systems increases, the adoption in practice would likely improve. Cow-attached sensors have been reported to reach a sensitivity of 90.2% and a specificity of 91.7% [14], whereas Van Nuffel et al. [15] reached an overall sensitivity of 88% and specificity of 87% using a walkover device. Using another type of walkover system, Bicalho et al. [7] achieved sensitivity and specificity results of 33.3% and 89.5%, respectively, whereas cameras have reached sensitivity values of 47.1–54.9% and a specificity of 90.4–94.1% [6]. The expected investment cost for these prototypes is unknown, but is presumably rather high because they are still in development and not yet fully adapted for use on a farm. Current prototypes seem to need improvement by improving the detection performance and lowering the system cost to be preferred over visual detection. This could be achieved by downscaling current system prototypes and by looking for new sensor-derived variables that may be useful to increase the performance of derived detection algorithms, as has been attempted for the Gaitwise walkover system by Van De Gucht et al. [16,17].

### 4.2. Farmers Are Willing to Pay for Improved Lameness Detection and Indication Which Leg Is Lame

WTP values indicated that farmers are willing to pay €2.57 more per % less missed lame cows and €1.56 more per % less false alerts. These values were rather high, but may be overestimated due to the fact that respondents are not bound by real-life constraints when making choices in a choice experiment [18]. Nevertheless, these WTP values give an indication to what extent the system cost can increase if the performance is improved to reach a similar usefulness for the farmer compared to the original system. But, WTP-values do not take into account how this usefulness is translated into actual purchase decisions and adoption rates. In other words, although usefulness may be the same for the improved version of the system, it may still not be high enough for farmers to adopt it in practice. Hence, costs for improvement should be lower than the willingness to pay in order to increase system usefulness.

Farmers were willing to spend an extra €12.7 per cow per year on a system capable of indicating which leg is lame compared to one that is not. Although farmers want to pay a substantial amount of money for this ability, its actual usefulness may be limited in practice. Lame cows tend to be trimmed on both their left and right hind hooves during claw trimming, which reduces the benefit of this ability. However, since lameness is situated on the front hooves in only 25% to 10% of the cases [19,20,21,22], front hooves are often not trimmed. Hence, indicating whether the problem is situated on a front or hind leg may be useful for the farmer. Furthermore, personal communication with farmers revealed that they may find this feature useful in the case of subclinical lameness. As the lame leg may not easily be identified and the actual lesion may still be hidden, the farmer may be unable to locate the lesion during a checkup. By indicating which leg is lame, the farmer or hoof trimmer has some prior knowledge about the affected leg and where to look in detail for possible problems. If the problem is not located on the hooves, such prior knowledge may facilitate diagnosis. 

### 4.3. Discrete Choice Experiments Provide Valuable Input for System Development

One of the goals of this study was to demonstrate how discrete choice experiments could be useful for system developers to improve their prototypes towards commercially viable, well-adopted systems. For example, developers could question whether the performance of a prototype should be increased or the cost reduced to optimally improve the prototype. If only system cost is altered, the desired combination is the intersection point of a vertical line (constant performance) through the point with the current characteristic levels and the desired utility plane (cfr. Plane 2 in Figure 1). For the example used in this study, altering the system cost only does not allow reaching the desired utility, since the vertical line through the current system (P1 in Figure 5) does not intersect with the desired utility planes (Plane 2 and 3 in Figure 5) within the range of possible values. Altering the detection performance by changing the percentage missed lame cows and the percentage false alerts while keeping system cost constant, allows one to reach the desired utility. In practice, it may be impossible to change only one characteristic to a large extent without deteriorating the level of the other system characteristics (e.g., decreasing system cost significantly without negatively affecting detection performance). This is caused by a system-specific relation between the system cost and performance on the design side, which technically limits the possible characteristic level combinations for the developer. As a result, large adaptations to the system will often require simultaneous alterations on the percentage missed lame cows, the percentage false alarms, and the system cost to reach the desired utility.

Two restrictions should be kept in mind when performing simulations using a utility model derived from DCEs. First, simulation values should lie within the range of possible system characteristic levels covered in the discrete choice experiment (Table 1). Second, the chosen system characteristic values and their combination should be realistic. In realistic scenarios, the varying detection performances of aforementioned prototypes could greatly influence market shares. Hence, the attribute levels of all systems—thus the other options—should be realistic to obtain realistic market shares. If not, the simulation results will not be realistic either, resulting in skewed market shares and unrealistic expectations. Keeping these restrictions in mind, the presented method provides the possibility to assess the potential adoption rates of completely different systems. For example, one could simulate the market share of a very cheap but low performant sensor system, but also a more expensive high performant system could be simulated and compared to the prototypes of other technologies. 

Besides the constraints of the utility model, a combination of system characteristic levels obtained using the method presented in Figure 5 might be technically or economically unrealistic (cfr. relation between system cost and performance). For example, if the development costs used to reach the desired performance become very high, or if the sensor hardware costs increase significantly, the system’s selling price may rise above the desired system cost. Hence, certain combinations of system characteristics may not be achievable for the developer. Consequently, development goals need adjustment to be more feasible while still reaching the desired usefulness for the farmer.

Farmers’ preferences for automatic lameness detection system characteristics vary, as they are influenced by certain socio-demographic characteristics such as the importance that the farmer attaches to lameness, and whether the farmer already uses an estrus detection system on the farm [12]. As such, different systems with specific characteristic levels might be more useful for different target groups. For example, farmers with a severe lameness problem in their herd may be better off using systems that focus on the detection of very lame cows in a first stage, and hence settle with a lower detection performance for mild lameness. Otherwise, the farmer would be inundated with lameness alerts and could lose the overview. Only once the severe lameness problems are under control, a detection system focusing on mild lame cows would be feasible for these farms. An alarm prioritization system may offer a solution to reduce the number of lame cow alerts and prevent the farmer from losing overview. Farmers that already have an efficient lameness management might need a more performant system to also detect mild lameness in an earlier stage. For those farms, lameness costs are already lower compared to farms with severe lameness problems. These farmers might aim for a reduction in labor time spent by performing visual lameness detection for practical or social reasons, and may hence be satisfied with a system that equals their own detection performance. Furthermore, farmer personality may influence the preferences for a detection system. In practice, some farmers may be willing to accept more false alerts if this implies no lame cow remains undetected. These farmers require a system with high sensitivity rather than high specificity. Other farmers may not be willing to accept false alerts and rather miss a lame cow than needlessly lose time to check up on a non-lame cow. The latter group requires systems with high specificity rather than high sensitivity. It would therefore be interesting if detection systems could be tailored to the farmer.

In practice, developers and manufacturers cannot easily develop different systems for different target groups, as this would entail high development and manufacturing costs. It could be better to aim for an average system performance that meets average farmer preferences and still reaches the desired market share for the manufacturer. Manufacturers could make this system adaptable according to the desires and daily management of the farmer by adjusting system settings, which could be done by changing detection thresholds for lameness alerts and by alert prioritization. This way, farmers could continue using the same system when the farm lameness status evolves from a severe lameness problem to a more acceptable situation thanks to better lameness detection and management. At the same time, the systems’ adaptability allows it to reach a high usefulness for many farmers, thus increasing the adoption rate of a one-fits-all automatic lameness detection system.

### 4.4. More Research on the Effect of Early Detection, Farm Scale and Economic Value of Automatic Detection Is Needed to Support Further Development and Allow More Accurate Market Share Estimations

Some system aspects that may be important for farmers have not been included in this study. The cost of automatic detection systems was incorporated in the model, but the economic value was not included as a result of unclear economic values of current system prototypes [11]. Early detection has been found to affect this economic value, and may be an important system characteristic for the farmer, as it was indeed important in studies concerning automatic mastitis detection preferences [23]. However, as farmers may currently delay the treatment of detected lameness in practice [24], early detection may only become important to farmers once they no longer underestimate lameness consequences and prevalence, as currently seems to be the case [25,26]. Little is known about how much earlier a detection system can detect lameness compared to the farmer [11], hence farmers’ preferences for early detection were not yet investigated in this study. Furthermore, as farmers were shown a system cost per cow in the choice sets, possible relationships between system cost and herd size (e.g., walkover and camera systems vs. cow-attached systems) have still to be accounted for. When developing a one-fits-all system, developers should compare the current system cost per cow for different herd sizes with farmers’ preferences to determine a maximum target system cost. More research is needed to investigate which other aspects related to detection system characteristics or the socio-demographic characteristics of farmers could influence potential market shares of automatic lameness detection systems.

## 5. Conclusions

We performed simulations using a utility model derived from a discrete choice experiment to evaluate the effects of dairy farmers’ preferences, system detection performance, and system cost on the potential market share of automatic lameness detection systems. The obtained results indicated that the market potential for cow-attached systems is the largest, as this technology was preferred over walkover and camera systems. To become more competitive and win more market share, the other systems should have higher detection performances or lower system costs. Reducing the system cost by €5 led to an average increase of 0.75% in the market share of each automatic detection system. Improving the detection performance by decreasing the percentage missed lame cows and percentage false alerts with 6% and 3%, respectively, led to 3% higher market shares on average for all automatic detection systems. The average willingness to pay was €2.57 per percent less missed lame cows, €1.65 per percent false alarms, and €12.7 for indication which leg is lame. A 3D presentation of the estimated utility values was proposed as a tool for a tailor-made and demand-driven technology design. Manufacturers and developers of automatic lameness detection systems could use the results of discrete choice experiments to estimate the adoption potential of their system and to assess which adjustments would result in the largest increase in the adoption potential. 

## Figures and Tables

**Table 1:** System characteristics and their respective possible levels used in the choice experiment reported in [12].

System Characteristics	Possible Characteristic Levels
Percentage missed lame cows	0%, 10%, 20%, 30%
Percentage false alarms	0%, 5%, 10%, 15%
Cost per cow per year	€15, €35, €55, €75
Indication lame leg	yes, no

**Table 2:** Example of a choice set as presented to the farmer. Only one option could be chosen.

Characteristics	Option 1	Option 2	Option 3	Option 4
	System Attached to the Cow	Walkover System	Camera System	Opt out
Percentage missed lame	10%	10%	30%	I will detect the lame cows myself using visual inspection
Percentage false alarms	15%	15%	0%	
Cost/cow/year	€55	€35	€15	
Indication lame leg	yes	no	yes	
